# Self-forming TiBN Nanocomposite Multilayer Coating Prepared by Pulse Cathode Arc Method

**DOI:** 10.1186/s11671-016-1564-9

**Published:** 2016-07-27

**Authors:** Yongzhi Cao, Zhenjiang Hu, Leilei Yan, Fuli Yu, Wendi Tu

**Affiliations:** Center for Precision Engineering, Harbin Institute of Technology, Harbin, 150001 People’s Republic of China

**Keywords:** TiBN, Self-forming multilayer, Nanocomposite, Cross-section, HRTEM

## Abstract

Novel multilayer structured TiBN coatings were deposited on Si (100) substrate using TiBN complex cathode plasma immersion ion implantation and deposition technique (PIIID). The coatings were characterized by X-ray diffraction (XRD), high-resolution transmission electron microcopy (HRTEM), energy-dispersive spectrometer (EDS) and ball-on-disk test. XRD results reveal that both samples of TiBN coatings have the main diffraction peak of TiN (200) and (220). Cross-section TEM images reveal that these coatings have the character of self-forming multilayer and consists of face-centered cubic TiN and hexagonal BN nanocrystalline embedded in amorphous matrix. Because of the existence of hexagonal BN, the friction coefficient of the new TiBN coating in room temperature is obviously lower than that of the monolithic TiN nanocrystalline coating.

## Background

For high-speed precision instruments in aerospace and other high-tech fields, reducing the heat yield of the device is the basis for work piece stability [1]. Surface modification technology can significantly improve the surface performance with micro size decals while containing mechanical properties of the matrix [2–4]. Solid lubrication materials, such as MoS_2_, V_2_O_5_, DLC, and WS_2_, have been developed and widely applied in satellite, spaceship, aerospace equipment, and the space station [5–11]. However, few of these coating systems have the capacity of containing low coefficient in a high-temperature condition [12], while more and more industrial parts especially high accuracy bearings require low friction performance under the condition of high temperature in the field of aerospace.

Previous studies in the literature are mainly using the method of increasing B element to the coatings and the main purpose is to form cubic BN (c-BN) or hexagonal TiB_2_ phases, which can effectively improve the hardness of the film [13, 14]. Some authors have synthesized TiBN/Ti(B)CN/WBN coatings with high hardness (≥30 GPa) for the reason of forming the main alloy phase of h-TiB_2_ or c-BN phase [15–18]. Because of its stable performance under high-temperature conditions and the good lubrication properties like graphite, h-BN has great potential in solid lubrication material development [19, 20]. Combined with these properties, we want to use the h-BN as the cathode directly and using the PIIID technology to form the TiBN coating including the phase of h-BN as the solid lubrication phase.

In this work, a novel TiBN nanocomposite multilayer TiBN coating containing nanoscale crystals was fabricated on a single silicon substrate by a PIIID technique. In order to characterize and understand the structure/composition relationship in the TiBN coating, the nanostructure and hexagon BN phase were examined by high-resolution transmission electron microcopy (HRTEM) in the newly developed coating, which have the capacity of containing low coefficient in high-temperature condition.

## Methods

Ternary TiBN coating was deposited onto polished single Si (100) substrates by using PIIID equipped with a TiBN complex cathode and is composed of the desired implanting and deposition material. Synthesis of the TiBN complex cathode target was conducted by powder metallurgy technology. The composition of the target with the weight percentage h-BN of 8, 30 to the Ti powder and then being forged under the pressure of 83 MPa and annealed at 1050 °C in a vacuum container. The base pressure in the chamber was 5 × 10^−3^ Pa, and the working pressure was 3 × 10^−1^ Pa during implantation and deposition. Synthesis of the TiBN coating was conducted in a flowing N_2_ gas, with an N_2_ ratio of 50 sccm. The substrate temperature was below 200 °C during the coating deposition. A pulsed bias with a voltage of 20 kV, a repetition frequency of 50 Hz, and a pulse duration time of 60 μs were applied on the substrate during the PIIID process. The parallel distance between the source and the substrate was 15 mm. The total implantation and deposition time was 4 h and the coating thickness was about 500 nm.

The as-deposited coatings were characterized by ultra-high-resolution transmission electron microscopy (TEM JEM-3000F) with an acceleration voltage of 300 keV, energy-dispersive spectrometer (EDS, Bruker EDS QUANTAX), and X-ray diffraction at a grazing angle of 1° (XRD, Empyrean) with a Cu-Kα radiation source at 40 kV and 40 mA. Specimens for cross-sectional TEM were prepared using the procedure of Helmerson and Sundgren [21]. The tribological properties of the TiBN coating were evaluated using a ball-on-disk tester with a linear velocity of 0.05 m/s. The tests were carried out under dry running conditions of a ф6.3 mm Si_3_N_4_ ball and a load of 30 g with the friction radius of 2 mm for 10 min. The revolution speed setting is 600 rpm.

## Results and Discussion

Table 1 shows the composition of TiBN nanocomposite coatings tested by EDS. It is apparent that the B content can be varied conveniently from 4.33 to 24.81 at.% by changing the h-BN ratio of TiBN complex cathode. We define the contents of TiBN complex cathodes with the weight percentage of 8 and 30 wt.% as samples a and b, respectively.

**Table 1:** Compositions of the as-deposited coatings as determined by EDS

Simple	Ti at.%	B at.%	N at.%	O at.%
a	41.60	4.33	43.71	10.36
b	18.46	24.81	46.94	9.79

**Fig. 1:**
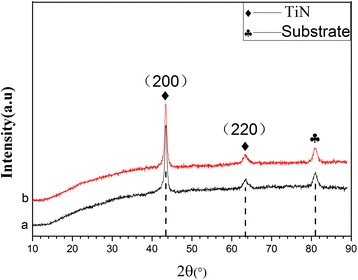
X-ray diffraction patterns of the as-deposited TiBN coatings with different BN contents. (*a*) 10 wt.%. (*b*) 30 wt.%

Figure 1 shows the XRD patterns of the nanocomposite coatings with different h-BN contents. According to Bragg angles (2θ) of 42.6° and 61.8° (JCPDS No. 38-1420), we can see that the XRD peaks of the Ti-B-N coating exhibits two characteristic TiN peaks of (200) and (220) planes of face-centered cubic (fcc) B1-NaCl-structured TiN. Both of the TiN peaks are shown to be offset on the right and with the increasing of BN content, the offset somewhat increased. We know that most of the coatings in the process of formation usually are inevitably understood by the compress stress in the inner of the coatings [22–26]. From the X-ray status, we confirm that the TiBN coatings withstand compressive stress, which result the peaks of TiN (200) and (220) shift to right and the radius of TiN to be decreased about 0.8 Å.

**Fig. 2:**
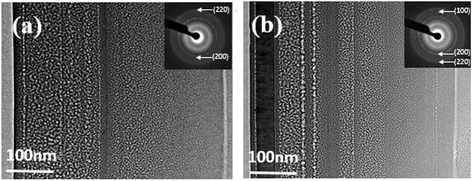
TEM cross-section micrographs of the TiBN composite coatings with different BN contents. **a** 10 wt.%. **b** 30 wt.%

Figure 2 shows the transmission electron microscopy (TEM) and corresponding diffraction rings of different h-BN content TiBN coatings. When the BN content is 8 wt.%, it is clear that the presence of a multilayer structure of layers of different thicknesses TiBN coating and there were only cubic TiN (200) and (220) plane exist in the multilayers. When the BN content is 30 wt.%, the multilayer structure is more obviously and the h-BN crystal face of (100) had formed in the outside of the TiBN coating.

**Fig. 3:**
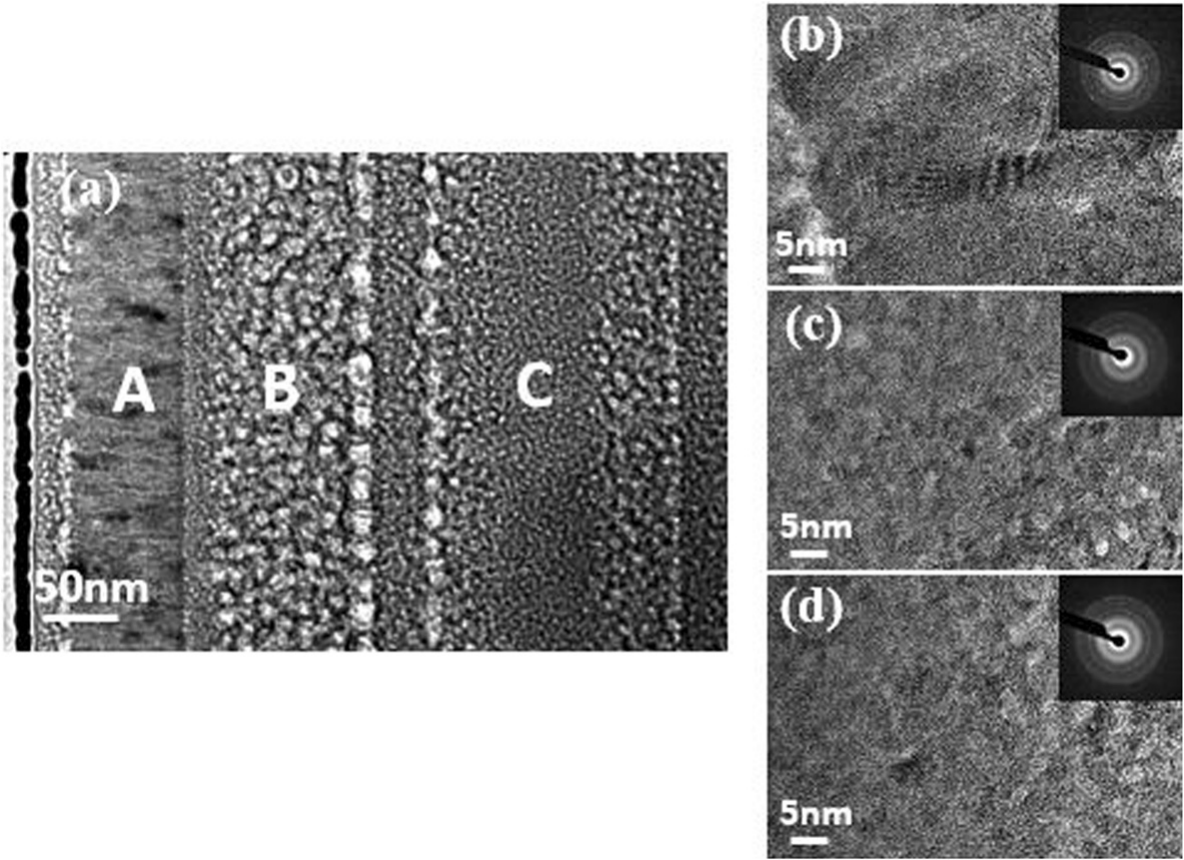
HRTEM images and SAD patterns of the TiBN composite coating. with 30 wt.% BN content. **a** TEM cross-section image. **b** HRTEM image of section *A* and corresponding SAD pattern. **c** HRTEM image of section *B* and corresponding SAD pattern. **d** HRTEM image of section *C* and corresponding SAD pattern

In order to identify the microstructure of the multilayer in the TiBN coating of 30 wt.% BN content, HRTEM images for different layers are shown in Fig. 3. The HRTEM images of sections A, B, and C are shown in Fig. 3b–d, and the selected area electron diffraction (SAD) result of each region is inserted into the respective image. From the result, we conclude that the h-BN may display a significant influence in the forming of TiBN coatings, when the content of h-BN is relatively lower in the plasma, the B^+^ ion were mainly act as the refine the grain of the TiN in the TiBN coatings, while with the addition of B^+^ ion in the plasma, the hexagonal BN phase has formed in the TiBN coatings.

At the process of the coating formation in the PIIID technology, ion bombardment the substrate when the pulse high voltage act on it, the sample temperature began to rise sharply, the crystallization temperature when the temperature reached after the film in the part of amorphous crystallization, so a part of the nanocrystalline, until the temperature balance, crystallization in balance, in addition, B ion doping will be very good fine grains. With the above reasons we know that the h-BN had double influence on the deposit the TiBN coatings.

**Fig. 4:**
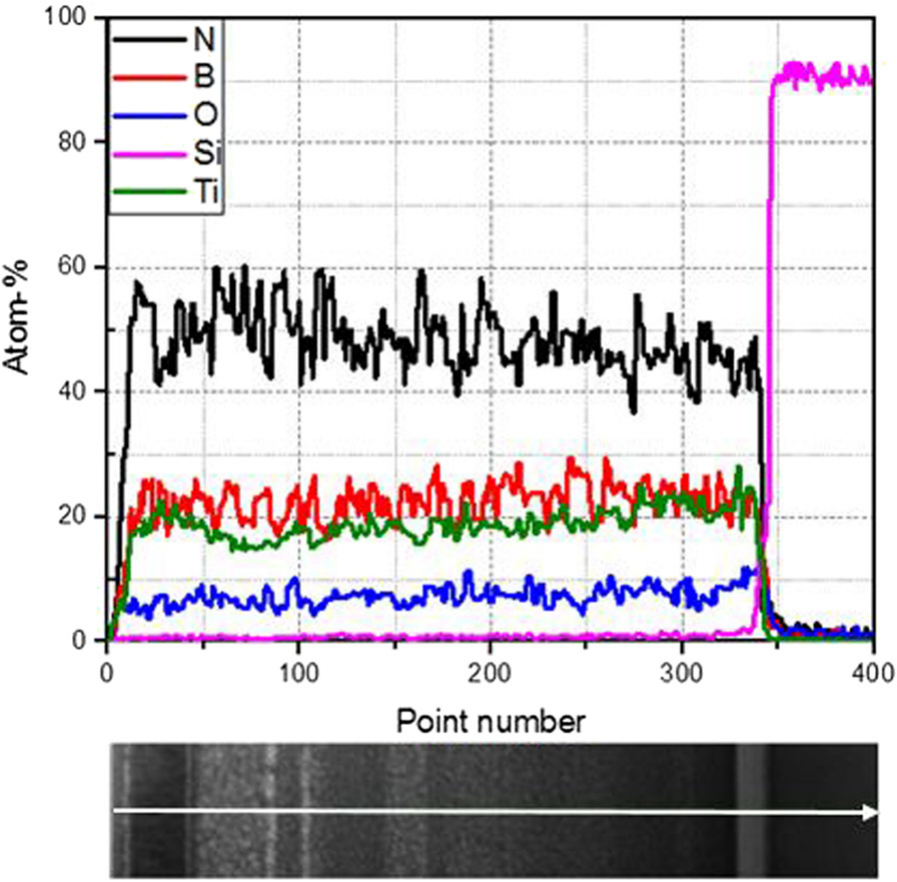
TEM pattern and EDS spectrums of TiBN coatings

The TEM cross-section and element line scanning of the TiBN coating are exhibited in Fig. 4. According to the line canning spectrums, the main elements of the coating are Ti, B, and N. In addition, obvious concentration fluctuations for these elements can be found, which hints that different structures may be formed in different depths. However, the mean concentrations for Ti, B, and N across the coating almost keep stable, which hints that the vacuum arc plasma kept stable in the deposition process.

**Fig. 5:**
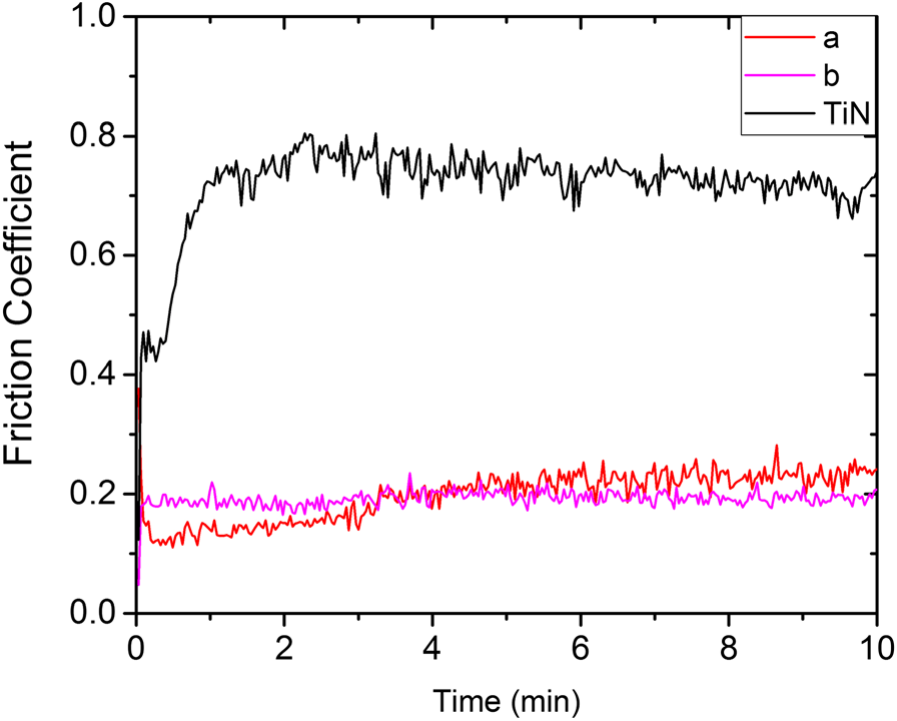
Friction curves of TiBN coatings with different BN contents at room temperature

Figure 5 shows the friction curves of the TiBN coatings at the room temperatures. We can see that the samples had the similar friction coefficient of ~0.2 after 10 min later when the film to be stable and form a stable scratch. The formation of solid lubrication film layer is conducive to the stability of the friction coefficient. As the increase of the content of h-BN, the coefficient of TiBN coatings has an obvious slow and decreased gradually at the friction test.

To render the coating low coefficient, the hexagonal BN added in the coating act as a solid lubricate that decrease shear band propagation in the adjacent layers while, at the same time, facilitating the formation of controlled shear bands within themselves. In addition, B+ can effectively refine the grain size and the density of film but also to the oxidation resistance of membrane and the improvement of the performance of the friction. Amorphous BN nanocrystalline layers play a part in enhancing the ductility of the TiBN coating. Consequently, the TiBN coating exhibits a unique combination of low coefficient in high temperature comparison to the monolithic TiN and TiAlN nanocrystalline coatings. It is anticipated that the newly developed coating could be used for aerospace equipment and the space station applications where high coefficient is not tolerated.

## Conclusions

In summary, a novel multilayer structured Ti-B-N coating is deposited on a single Si (100) wafer substrate by a PIIID technique. The newly developed coating consists of vertically aligned, alternating nanocrystalline TiN, BN, and amorphous layers. It exhibits low coefficient in room-temperature conditions, due to the hexagonal BN phase and nanocomplex composition structure. The improved performance was found to be derived from the self-forming multilayer properties and the h-BN solid lubrication effects in the coating.
